# Effects of implantation of bone marrow cells on cytokine levels in the ischemic heart tissue. An experimental study

**DOI:** 10.1186/1749-8090-3-30

**Published:** 2008-05-20

**Authors:** Yahya Unlu, Sami Karapolat

**Affiliations:** 1 Department of Cardiovascular Surgery, Faculty of Medicine, Ataturk University, Erzurum, Turkey; 2 Menderes Cadd, No: 52/8, Buca, Izmir, Turkey

## Abstract

**Background:**

In order to achieve a safe and persistent angiogenic effect, we investigated the potential of bone marrow cells implantation to enhance angiogenesis of ischemic hearts in a rat model, and also we have investigated growth factors accompanying and intermediating the angiogenesis, and the changes occurring in the levels of cytokines and their relations with angiogenesis.

**Methods:**

30 adult male Wistar albino rats from the same colony were used. After anterior myocardial infarction induced by occlusion of the left anterior descending artery, they were divided into two groups (Group I and Group II). 2 × 10^7 ^bone marrow cells suspended in 0.1 ml phosphate-buffered saline solution and 0.1 ml phosphate-buffered saline solution were injected at six points in the infarcted area in Group I and Group II respectively. Changes in the vascular density and, vascular endothelial growth factor, vascular cell adhesion molecule and cytokine levels in the infarcted myocardium after bone marrow cells implantation were examined.

**Results:**

The implantation assay showed that bone marrow cells induced angiogenesis. Light microscopic analysis of the vascular density in the ischemic area showed that, angiogenesis had been induced to higher in Group I than Group II. Levels of vascular endothelial growth factor, vascular cell adhesion molecule and the inflammatory cytokines such as interleukin-1 and tumor necrosis factor-α in Group I were significantly elevated compared with those in Group II.

**Conclusion:**

Bone marrow cells implantation induced angiogenesis in a rat ischemic heart model as a result of increase of the levels of vascular endothelial growth factor, vascular cell adhesion molecule, interleukin-1, and tumor necrosis factor-α.

## 1. Introduction

Although medical therapy and coronary revascularization techniques such as percutaneous balloon angioplasty and stenting or surgical procedures improve the prognosis and survey on coronary artery disease, a substantial number of patients are failed despite maximal conventional therapy because of not being suitable for coronary revascularization. Improvement of neovascularization techniques attenuates myocardial ischemia in coronary artery disease. In order to promote neovascularization, several therapeutic strategies have been developed including the addition of angiogenic growth factors [1,2].

Cell transplantation is a novel therapeutic option for myocardial repair in hearts with postinfarction congestive heart failure, unreconstructable coronary atherosclerosis, or cardiomyopathy [3-5]. Implantation of nonselected bone marrow cells into the ischemic myocardium has been utilized to treat these patients. Bone marrow cells provide angiogenic precursors and angiogenic cytokine-producing cells in myocardium. Also, erythroid cells are essential for the in vivo effects of bone marrow cell implantation. In addition, bone marrow cells are a source of multiple growth factors involved in neovascularization, including vascular endothelial growth factor (VEGF).

Therapeutic angiogenesis describes an emerging field of cardiovascular medicine whereby new blood vessel growth is induced to supply oxygen and nutrients to ischemic cardiac or skeletal muscle [6,7]. The growth of this field has exploded in the past decade as a result of the development of recombinant growth factors, the best characterized of which is the soluble mediators' basic fibroblast growth factor and VEGF. Both of these factors stimulate in vivo angiogenesis [7,8], and numerous preclinical studies utilizing protein therapy in a variety of animal models have demonstrated improvements in perfusion, function, and vascularity [7,9].

Several chemokines and cytokines have been shown to promote mobilization of hematopoietic stem cells and endothelial progenitor cells. Mobilization by granulocyte-colony stimulating factor is achieved by the disruption of the homing mechanisms of stem cells in the bone marrow, e.g. by proteolytic cleavage of vascular cell adhesion molecule (VCAM) [2,10]. Also, sVCAM has been reported to exhibit angiogenic activity in vivo through mediating endothelial cell chemotaxis activity [11]. In experimental models, mobilization of stem cells was also achieved by injections of chemokines such as interleukin-1 (IL-1β) and tumor necrosis factor-α (TNF-α) [2,12-14].

In this study, we designed a rat myocardial ischemia model to investigate the angiogenic ability of implanting bone marrow cells in an acute myocardial infarction model, and attempted to elucidate the possible mechanism of neovascularization.

## 2. Materials and methods

### 2.1. Rats

30 adult male Wistar albino rats with an average of 200–250 g body weight from the same colony were used. The purpose of using rats is easy availability, safety and the high ratio of repeating the experiment and because of their minimal myocardial collaterals. The experiments were conducted in accordance with the Guidelines for the Care and Use of Laboratory Animals published by the National Institutes of Health (NIH Publication No. 85-23, revised 1996). They were kept at 21° to 23°C, with controlled humidity, and a dark-light cycle of 12 to 12 h. Food and water were available ad libitum. The experimental protocol was approved by Ataturk University, School of Medicine, Animal Care and Use Committee.

### 2.2. Preparation of rat bone marrow cells

Randomly selected 5 rats were sacrificed and bone marrow from the femur and tibia was collected and a total of 4 ml of bone marrow blood was placed in phosphate-buffered saline (PBS). Bone marrow cells were washed twice in PBS and suspension was prepared through a fine wire mesh. An optimal number of cells (2 × 10^7^) were suspended in 0.1 ml PBS.

### 2.3. Rat ischemic heart model

Rats were anesthetized with 50 mg/kg pentobarbital intraperitoneally. After tracheostomy, rats were entubed with 16G catheter. Rats were given ventilator support using room atmosphere in volume control mode throughout operation. 90/minute respiration count and 15–20 ml/kg minute volume were provided during ventilation (Hugo sacs, rodent ventilator, Germany). Reason of this, mediastinal pleura is not complete and is quite weak in rats. Unless a positive pressure ventilation is achieved, pleura is sutured and both lungs collapse even when only one pleura is opened.

In operation, rats were put into right posterolateral position; the site was shaved and disinfected by using povidone iodine. A left thoracotomy was performed through the fourth intercostal space and the left anterior descending coronary artery (LAD) was ligated with 7.0 silk suture. The rats were then randomly divided into two groups after LAD ligation. In Group I (n = 12), the rats were injected, at six points in the infarcted area using a 26-gauge needle (2 × 10^7 ^bone marrow cells suspended in 0.1 ml PBS). In Group II (n = 13, control group), the rats were injected, at six points in the infarcted area using a 26-gauge needle (0.1 ml PBS). Injections are made at the end of about a fifteen-minute-waiting-duration following the ligation of LAD. The detection of infarcted area by the observation of the discoloration formed in the left ventricular anterior wall macroscopically. In general, it was determined that the infarcted area was about 3 × 2 mm^2^and that the injection sites with a 1 mm distance among them were distributed homogenously throughout the infracted area. The size of the infarcted area formed enables 6 different injection application points, and thus depending on the size of infarcted area, direct intramyocardial injections were applied from the six points of the infarcted area and bordering zone. These injections were successfully performed without any acute complication.

Then, bleeding control was done, the lung was expanded and thoracotomy incisions were closed properly. When the spontaneous respiration was achieved, rats were extubed, tracheostomy was closed and they were kept in different cages. In the first 24 hours rats were given Buprenorphine 0.03 mg/kg every 8 hours subcutaneously. Antibiotic therapy with cefazolin sodium (15 mg/kg/intramuscular) was given for 10 days. They were given food and water as much as they wanted. Wound infection developed in one rat (in Group II) and excluded from the experiment protocol.

### 2.4. Changes in the levels of VEGF, IL-1β, sVCAM, and TNF-α

On the 1^st ^day after the operation, 2 rats both from the two groups, on the 3^rd ^day 2 rats both from the two groups and on the 5^th ^day 3 rats both from the two groups were sacrificed by administering lethal Ketamine intraperitoneally. The hearts were extracted and samples of the myocardial infarct area were collected. All samples were minced and homogenized in PBS on ice, then centrifuged at 10,000 g for 5 min at 4°C, and the resulting supernatants were stored at -80°C. Vascular endothelial growth factor (VEGF), interleukin-1 (IL-1β), soluble vascular cell adhesion molecule (sVCAM), and tumor necrosis factor-α (TNF-α) in supernatant fluid were determined by a standard enzyme-linked immunoabsorbent assay method (VEGF, IL-1β, sVCAM, and TNF-α ELISA kits, R&D systems, USA) according to the instructions of the manufacturers. The levels of these growth factors and cytokines were expressed as picograms of VEGF, IL-1β, and TNF-α and, as nanograms of sVCAM per tissue wet weight and used for statistical analysis.

### 2.5. Evaluation of angiogenesis induced by the implantation of bone marrow cells

In the 1^st ^week after the operation, 1 rat each both from the two groups, in the 2^nd ^week 2 rats each both from the two groups, and in the 4^th ^week 2 rats from Group I and 3 rats from Group II were sacrificed by administering lethal Ketamine intraperitoneally. The hearts were extracted and samples of the myocardial infarct area containing marginal and normal areas were embedded in OCT compound, snap-frozen in liquid nitrogen, and cut into 5-μm-thick sections. Tissue sections were immunostained with mouse anti-rat CD31 monoclonal antibody using the Dako LSAB 2 Kit, Peroxidase for use on rat specimens. The sections were fixed with 99% acetone for 10 min at 4°C, 3% hydrogen peroxide was applied to cover each specimen to block endogenous peroxidase activity for 5 min. The sections were incubated with mouse anti-rat CD31 antibody at 1:200 dilutions for 30 min at room temperature, and developed with 3.3'-diaminobenzidine plus nickel sulfate in phosphate buffer-H_2_O_2_. Counterstaining was performed with methyl green. Angiogenesis was assessed by counting the number of vessels under light microscopy. The entire infracted area was examined under ×200 microscopy to determine the mean number of vessels per square millimeter.

### 2.6. Statistical analysis

The results were evaluated statistically. All data are presented as the mean ± standard error. One-way between-groups analysis of variance (ANOVA) was used to compare VEGF, IL-1β, sVCAM, and TNF-α between groups. Statistically p > 0.05 was accepted as insignificant, and p < 0.05 as significant.

## 3. Results

In this study; neovascularization, as defined by density of microvessels and VEGF, IL-1β, sVCAM, and TNF-α levels in the infarcted myocardial tissues was quantified in the Group I and Group II.

### 3.1. VEGF, sVCAM and cytokine levels in the infarcted myocardium after implantation of bone marrow cells

**Figure 1:**
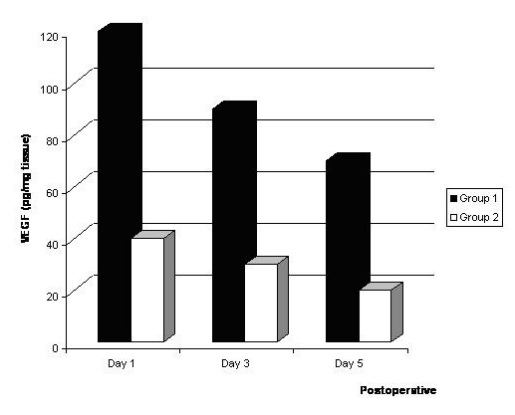
Levels of VEGF* in the infracted myocardium after bone marrow cell implantation. *The levels of VEGF, IL-1β, TNF-α (pg/mg) and sVCAM (ng/mg) in the restricted infracted myocardium per wet weight are indicated.

The changes in VEGF levels are shown in Fig 1. A significantly higher VEGF level in the infarcted myocardial tissues was observed in the Group I than Group II.

**Figure 2:**
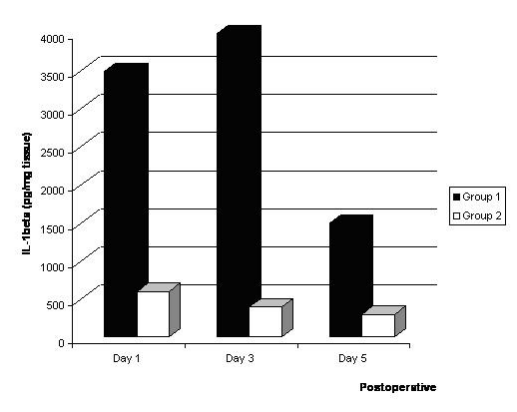
Levels of IL-1β* in the infracted myocardium after bone marrow cell implantation. *: The levels of VEGF, IL-1β, TNF-α (pg/mg) and sVCAM (ng/mg) in the restricted infracted myocardium per wet weight are indicated.

**Figure 3:**
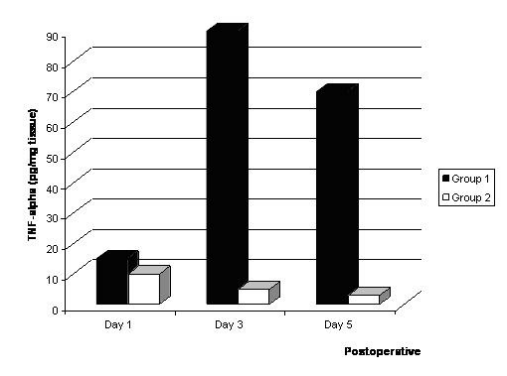
Levels of TNF-α* in the infracted myocardium after bone marrow cell implantation. *: The levels of VEGF, IL-1β, TNF-α (pg/mg) and sVCAM (ng/mg) in the restricted infracted myocardium per wet weight are indicated.

As shown in Fig 2 and Fig 3, the concentrations of IL-1β and TNF-α in the infarcted myocardial tissues were significantly higher in the Group I than Group II.

**Figure 4:**
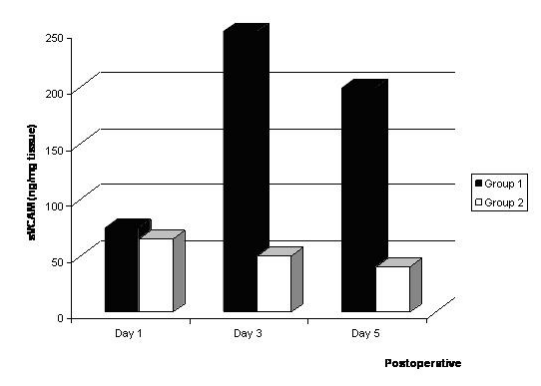
Levels of sVCAM* in the infracted myocardium after bone marrow cell implantation. *: The levels of VEGF, IL-1β, TNF-α (pg/mg) and sVCAM (ng/mg) in the restricted infracted myocardium per wet weight are indicated.

Furthermore, the concentrations of sVCAM in the infarcted myocardial tissues were also significantly higher in the Group I than Group II (Fig 4).

Myocardial expressions of VEGF(61.1 ± 4.4 vs. 16.2 ± 3.4, p < 0.01), IL-1β (1240.1 ± 12.1 vs. 213.2 ± 13.2, p < 0.01), TNF-α (65.3 ± 4.5 vs. 2.1 ± 2.4, p < 0.01), and sVCAM (156.3 ± 3.8 vs. 32.6 ± 4.2, p < 0.01) levels in implantation of bone marrow cells into the infarcted myocardial tissues was significantly higher than that of Group II 5 days after treatment.

### 3.2. Determination of angiogenesis by histopathological examination

The numbers of vessels in per 5 square millimeter fields of each histopathological preparation prepared separately for each subject were calculated and in the end, individual means of these values were found for each group. At the end of the 1 week, the mean number of vessels in per 5 square millimeter fields was found to be 24.0 ± 4.2 in Group I, and 13.3 ± 3.0 in Group II (p > 0.05). At the end of the 2 weeks, the mean number of vessels in per 5 square millimeter fields was found to be 46.4 ± 5.3 in Group I, and 19.7 ± 3.4 in Group II (p < 0.05). And, at the end of the 4 weeks, the mean number of vessels in per 5 square millimeter fields was found to be 61.4 ± 5.6 in Group I, and 23.2 ± 4.1 in Group II (p < 0.05).

In addition, angiogenesis was significantly increased in the center of the scar in Group I compared to Group II. As a result, the density of microvessels in the infarcted myocardial tissues was significantly higher in Group I than Group II, especially 2 and 4 weeks after the operation.

## 4. Discussion

In this experimental model, we demonstrated induced myocardial angiogenesis as evidenced by increased microvessels density. We observed that, intramyocardial implantation of bone marrow cells induced angiogenesis.

Replacement and regeneration of functional cardiac muscle after an ischemic insult to the heart could be achieved by either stimulating proliferation of endogenous mature cardiomyocytes, resident cardiac stem cells or by implanting exogenous donor-derived or allogenic cardiomyocytes. Angiogenesis was induced by the proliferation and migration of endothelial cells from existing vessels in adults [15-17]. A variety of growth factors are known to induce angiogenesis. VEGF plays an important role in the angiogenic process and promotes the proliferation and migration of endothelial cells specifically [18-20]. In the present study, we selected bone marrow cells as the material for inducing angiogenesis because they contain endothelial progenitor cells that can participate in vascular formation in severe ischemic lesions [21].

Bone marrow cells were considered optimal for implantation in this ischemic heart model. We found that a significant improvement in angiogenesis occurred within 2 weeks after implantation of bone marrow cells treatment. The capillary density in the Group I was found to be significantly increased 4 weeks after implantation of bone marrow cells. In this time, bone marrow cells treatment induced approximately 2.5-fold increase in capillary density as well.

Takahashi et al. reported that the implantation of bone marrow cells into ischemic hearts can induce angiogenesis by increasing the microvessel density and decreasing the fibrotic area in the infarcted myocardium significantly and improve cardiac function after myocardial infarction, and concluded that various cytokines are produced by bone marrow cells and these cytokines contributed to functional improvement of the infarcted heart by directly preserving the contractile capacity of the myocardium, inhibiting apoptosis of cardiomyocytes, and inducing therapeutic angiogenesis [22].

Recent studies have demonstrated that, the protein expression of inflammatory cytokines, such as IL-1β and cytocine-induced neutrophil chemoattractant, which have been proven to have angiogenic potency in vivo, was significantly increased in the implantation of bone marrow cells group compared with the other groups [[19,23], and [24]]. The main potential source for these inflammatory cytokines is monocytes, macrophages, and granulocytes. Bone marrow cells contain all these cells which have the ability to release inflammatory cytokines. TNF-α that one of the cytokines released from macrophages that induces angiogenesis [14,25], was detectable in this model.

In a study carried out by Tse et al., autologous bone marrow cell implantation was applied into the 12 patients with severe coronary artery disease who underwent electromechanical mapping-guided catheter-based. As a result, they found that bone marrow cell implantation into the ischemic human myocardium procedure is not associated with long-term major adverse effects such as tumor, scar, or calcification formation, and that the arrhythmogenic risk is low [26].

Although implantation of bone marrow cells is an invasive attempt, it increases the formation of microcapillary arteries and collateral neovascularization in infracted hearts, and improves ventricular heart function. Besides, it proliferates and activates myocardial cells in infarcted area. Thus, it decreases the infarct size and affects the postinfarction remodeling beneficially. As a result, an improvement in symptoms and exercise capacity, increase in myocardial perfusion and function at the ischemic myocardium occurs. Recently, this treatment protocol is seen as a promising, safe, effective and feasible method in ischemic heart diseases in terms of restoring tissue viability and increasing functioning of patients. From the point of effective administering way, Hayashi et al. found that bone marrow cells are more effectively delivered intramyocardially than intravenously for repairing injured myocardium [27].

The present study is established that bone marrow cells were effective for inducing therapeutic angiogenesis in a rat acute myocardial infarction model, the mechanism of which related to an increase in inflammatory cytokines, such as IL-1β, TNF-α and sVCAM. The implantation of bone marrow cells is a simple method for inducing angiogenesis without causing toxicity or immunological rejection, in comparison with other therapeutic methods of angiogenesis using human recombinant protein, naked plasmid DNA, and viruses. However, angiogenesis induced by whole bone marrow cell implantation was not dose dependent in this experiment, which may be explained in two ways. First, whole bone marrow cells consist of a variety of cells which may release a number of growth factors or cytokines, including promotive and inhibitive ones. Second, bone marrow cells may play a different role in the environment of ischemic myocardium or acute infarcted myocardium, and it is possible that stronger angiogenesis is induced by the implantation of bone marrow cells in ischemic myocardium, where they survive longer and are stimulated to secrete more angiogenic factors such as VEGF. The ischemic environment may facilitate the differentiation of endothelial progenitor cells in bone marrow to endothelial cells. Further investigations on implantation of bone marrow cells treatment are required to clarify the optimal populations of whole bone marrow cells that will have the most angiogenic potency and to determine whether implantation of bone marrow cells will induce stronger angiogenesis in an ischemic environment.

In an experimental study similar to our study by Nishida et al., it was found that bone marrow cell implantation treatment in hypoperfusion heart model formed in rats increased the microvessel density, blood flow and thickness of the left ventricular anterior walls significantly. As a result, they indicated that such an application induces angiogenesis and improves the perfusion of ischemic myocardium, and thereby prevents left ventricular remodeling and improves deteriorated cardiac function caused by myocardial hypoperfusion, and all these findings are in accordance with the results we obtained [28]. In general, it is the infarct size that determines the long-term prognosis of the patients after myocardial infarction. Bone marrow cell implantation into ischemic heart can repair the scar area and recover the myocardial function of the patients and thus improve the life quality by means of the above mentioned positive effects.

## 5. Conclusion

These results indicate that implantation of bone marrow cells can successfully promote neovascularization using a rat ischemic heart model. Also, the mechanism of this effect seems to be related to an increase IL-1β, sVCAM, and TNF-α. Our results proved that implantation of bone marrow cells in the ischemic heart tissue is safe and effective and, has implications for clinical treatments of patients with atherosclerotic coronary artery disease, and "end stage" cardiac disease. When the improvement in living comfort of the patients is taken into account, this treatment protocol is a choice to be tried in necessary situations. Nevertheless, all these views should be arranged in terms of the late period follow-up results to be obtained from further randomized, controlled studies on human.

## Competing interests

The authors declare that they have no competing interests.

## References

[B1] Isner JM, Asahara T (1999). Angiogenesis and vasculogenesis as therapeutic strategies for postnatal neovascularization. J Clin Invest.

[B2] Walter DH, Dimmeler S (2002). Endothelial progenitor cells: regulation and contribution to adult neovascularization. Herz.

[B3] Yau TM, Tomita S, Weisel RD, Jia ZQ, Tumiati LC, Mickle DA, Li RK (2003). Beneficial effect of autologous cell transplantation on infracted heart function: comparison between bone marrow stromal cells and heart cells. Ann Thorac Surg.

[B4] Yoo KJ, Li RK, Weisel RD, Mickle DA, Jia ZQ, Kim EJ, Tomita S, Yau TM (2000). Heart cell transplantation improves heart function in dilated cardiomyopathic hamsters. Circulation.

[B5] Yau TM, Li G, Weisel RD, Reheman A, Jia ZQ, Mickle DA, Li RK (2004). Vascular endothelial growth factor transgene expression in cell-transplanted hearts. J Thorac Cardiovasc Surg.

[B6] Hughes GC, Post MJ, Simons M, Annex BH (2003). Translational physiology: porcine models of human coronary artery disease: implications for preclinical trials of therapeutic angiogenesis. J Appl Physiol.

[B7] Hughes GC, Biswas SS, Yin B, Coleman RE, DeGrado TR, Landolfo CK, Lowe JE, Annex BH, Landolfo KP (2004). Therapeutic angiogenesis in chronically ischemic porcine myocardium: comparative effects of bFGF and VEGF. Ann Thorac Surg.

[B8] Papetti M, Herman IM (2002). Mechanisms of normal and tumor-derived angiogenesis. Am J Physiol Cell Physiol.

[B9] Tang YL, Zhao Q, Zhang YC, Cheng L, Liu M, Shi J, Yang YZ, Pan C, Ge J, Philips MI (2004). Autologous mesenchymal stem cell transplantation induce VEGF and neovascularization in ischemic myocardium. Regul Pept.

[B10] Levesque JP, Takamatsu Y, Nilsson SK, Haylock DN, Simmons PJ (2001). Vascular cell adhesion molecule-1 (CD106) is cleaved by neutrophil proteases in the bone marrow following hematopoietic progenitor cell mobilization by granulocyte colony-stimulating factor. Blood.

[B11] Koch AE, Halloran MM, Haskell CJ, Shah MR, Polverini PJ (1995). Angiogenesis mediated by soluble forms of E-selectin and vascular cell adhesion molecule-1. Nature.

[B12] Pruijt JF, Verzaal P, van Os R, de Kruijf EJ, van Schie ML, Mantovani A, Vecchi A, Lindley IJ, Willemze R, Starckx S, Opdenakker G, Fibbe WE (2002). Neutrophils are indispensable for hematopoietic stem cell mobilization induced by interleukin-8 in mice. Proc Natl Acad Sci USA.

[B13] Sweeney EA, Priestley GV, Nakamoto B, Collins RG, Beaudet AL, Papayannopoulou T (2000). Mobilization of stem/progenitor cells by sulfated polysaccharides does not require selectin presence. Proc Natl Acad Sci USA.

[B14] Heba G, Krzeminski T, Porc M, Grzyb J, Ratajska A, Dembinska-Kiec A (2001). The time course of tumor necrosis factor-alpha, inducible nitric oxide synthase and vascular endothelial growth factor expression in an experimental model of chronic myocardial infarction in rats. J Vasc Res.

[B15] Risau W (1997). Mechanisms of angiogenesis. Nature.

[B16] Schwartz Y, Kornowski R (2003). Progenitor and embryonic stem cell transplantation for myocardial angiogenesis and functional restoration. Eur Heart J.

[B17] Fujii H, Tomita S, Nakatani T, Fukuhara S, Hanatani A, Ohtsu Y, Ishida M, Yutani C, Miyatake K, Kitamura S (2004). A novel application of myocardial contrast echocardiography to evaluate angiogenesis by autologous bone marrow cell transplantation in chronic ischemic pig model. J Am Coll Cardiol.

[B18] Ferrara N, Davis-Smyth T (1997). The biology of vascular endothelial growth factor. Endocr Rev.

[B19] Kobayashi T, Hamano K, Li TS, Katoh T, Kobayashi S, Matsuzaki M, Esato K (2000). Enhancement of angiogenesis by the implantation of self bone marrow cells in a rat ischemic heart model. J Surg Res.

[B20] Itescu S, Schuster MD, Kocher AA (2003). New directions in strategies using cell therapy for heart disease. J Mol Med.

[B21] Asahara T, Murohara T, Sullivan A, Silver M, Zee R van der, Li T, Witzenbichler B, Schatteman G, Isner JM (1997). Isolation of putative progenitor endothelial cells for angiogenesis.. Science.

[B22] Takahashi M, Li TS, Suzuki R, Kobayashi T, Ito H, Ikeda Y, Matsuzaki M, Hamano K (2006). Cytokines produced by bone marrow cells can contribute to functional improvement of the infarcted heart by protecting cardiomyocytes from ischemic injury. Am J Physiol Heart Circ Physiol.

[B23] Giulian D, Woodward J, Young DG, Krebs JF, Lachman LB (1988). Interleukin-1 injected into mammalian brain stimulates astrogliosis and neovascularization. J Neurosci.

[B24] Yoshida A, Yoshida S, Hata Y, Khalil AK, Ishibashi T, Inomata H (1998). The role of NF-kappaB in retinal neovascularization in the rat. Possible involvement of cytokine-induced neutrophil chemoattractant (CINC), a member of the interleukin-8 family. J Histochem Cytochem.

[B25] Leibovich SJ, Polverini PJ, Shepard HM, Wiseman DM, Shively V, Nuseir N (1987). Macrophage-induced angiogenesis is mediated by tumor necrosis factor-alpha. Nature.

[B26] Tse HF, Thambar S, Kwong YL, Rowlings P, Bellamy G, McCrohon J, Bastian B, Chan JK, Lo G, Ho CL, Lau CP (2006). Safety of catheter-based intramyocardial autologous bone marrow cells implantation for therapeutic angiogenesis. Am J Cardiol.

[B27] Hayashi M, Li TS, Ito H, Mikamo A, Hamano K (2004). Comparison of intramyocardial and intravenous routes of delivering bone marrow cells for the treatment of ischemic heart disease: an experimental study. Cell Transplant.

[B28] Nishida M, Li TS, Hirata K, Yano M, Matsuzaki M, Hamano K (2003). Improvement of cardiac function by bone marrow cell implantation in a rat hypoperfusion heart model. Ann Thorac Surg.

